# Experts and end users discuss best practices and obstacles for deploying PM_2.5_ sensors in scientific, community, and educational settings

**DOI:** 10.1016/j.isci.2026.115333

**Published:** 2026-03-11

**Authors:** Kristen Okorn, Juthi Rani Mitra, Margaret Pippin, Kevin Czajkowski, Frank Marsik, Sara Mierzwiak

**Affiliations:** 1 Atmospheric Science Branch, NASA Ames Research Center, Moffett Field, CA 94035, USA; 2 Department of Geography and Planning, University of Toledo, Toledo, OH 43606, USA; 3 Chemistry and Dynamics Branch, NASA Langley Research Center, Hampton, VA 23681, USA; 4 Department of Climate and Space Sciences and Engineering, University of Michigan, Ann Arbor, MI 48109, USA

**Keywords:** Analytical chemistry, Sensor, Atmospheric science, Atmospheric observation, Education

## Abstract

This document reflects the combined perspectives of scientists in the low-cost sensor (LCS) field and Global Learning and Observations to Benefit the Environment (GLOBE) community organizers and educators for a holistic approach to measuring PM_2.5_ across disciplines and applications. These perspectives were shared during the virtual NASA GLOBE PM_2.5_ Low-Cost Sensor Workshop, May 7–9, 2025, through a series of short presentations and topical breakout room conversations. Presentations outlined successful advances in calibration, long-term deployments, satellite and remote sensing applications, and educational tools for communicating data from LCS. Subsequent discussion sections allowed educators, community members, and scientists to engage in meaningful dialogue about their successes and struggles in using LCS and their data. The needs of different end users are thoroughly explored to present best practices for several key applications and to present a set of recommendations to better bridge the gap between scientists and community partners to extract more useful data from the thousands of low-cost PM_2.5_ sensors deployed globally.

## Graphical abstract

**Figure:**
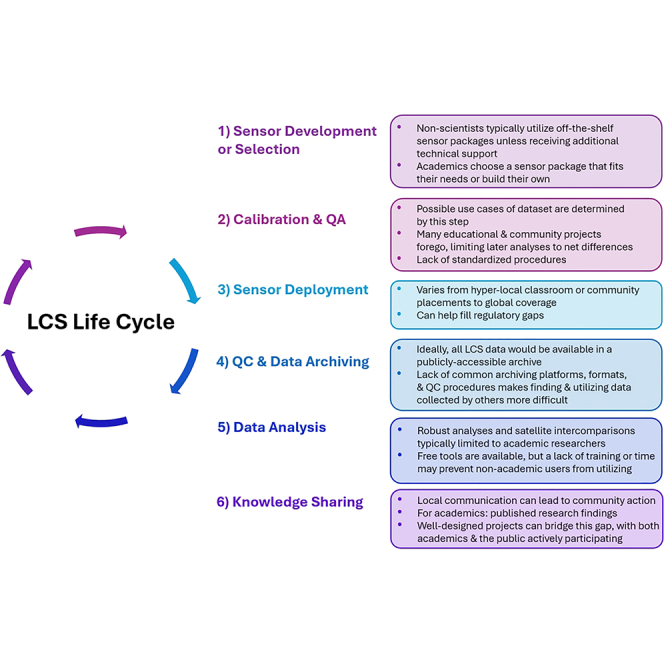

## Introduction

### Growth and challenges for low-cost sensors

While 94% of the global population is exposed to average annual PM_2.5_ concentrations deemed unsafe by the World Health Organization,^1^ disparities based on demographic and geographic factors have created unequal access to air quality (AQ) monitoring.^2^ In response, low-cost sensors (LCSs) have exploded in popularity over the past 15 years, offering real-time AQ data with high spatial and temporal resolution at a much lower upfront cost than traditional monitoring techniques. This has made LCSs a democratized platform for gathering AQ data across the globe, from large urban centers and underserved areas alike.

Prior to their advent, the majority of AQ monitoring was government-run and funded. In the years since, a 2018 assessment of LCS projects found that a notable amount utilized commercial or crowd-funded monitoring devices.^3^ As of 2025, The PurpleAir commercial LCS network alone boasts over 35,000 deployed PM_2.5_ sensors, offering global coverage.^4^ This widespread adoption of LCSs has brought AQ monitoring to innumerable areas that lacked government or regulatory AQ inventories previously, while technical leaps in sensor calibration and validation in the scientific space have improved the data quality of some LCSs. Specifically, addressing PM_2.5_ LCS’ sensitivity to environmental conditions such as humidity and degradation over time^5^ has afforded greater accuracy and precision.

However, these innovations have not necessarily been adopted or replicated by the majority of LCS end users, creating a large knowledge gap between researchers and most other end users.^6^ Unlike federal reference methods (FRMs) or federal equivalent methods (FEMs), there are no universal quality standards for LCSs. Validation and calibration are necessary to ensure accuracy and precision^7^ but may not be carried out by end users due to a variety of factors discussed in barriers to utilizing LCS and their data and calibration and QA/QC procedures. Addressing non-technical factors including end-user engagement and socioeconomic context could facilitate stronger knowledge transfer between professionals and the public.

### The GLOBE program and NASA GLOBE PM_2.5_ workshop

Established as a citizen science program in 1994, the Global Learning and Observations to Benefit the Environment (GLOBE) program engages the public to collect and analyze data, enhancing their understanding of the interconnected earth systems. The GLOBE program has a presence in over 127 countries, where scientist-developed “protocols” on the atmosphere, biosphere, hydrosphere, and pedosphere guide citizen scientists in collecting data by following standardized and scientific data collection procedures.^8^ These protocols are also used to support learning in primary and secondary (K-12 in the US) educational settings; GLOBE teachers receive formal training and educational resources to guide their students in conducting earth science research and reporting their findings. In addition to hands-on learning, GLOBE currently offers more than 270 million open access measurements.^9^ In an effort to expand their monitoring and outreach efforts into the LCS and specifically PM_2.5_ space, GLOBE co-hosted the NASA GLOBE PM_2.5_ Low-Cost Sensor Workshop from May 7–9, 2025.

**Figure 1:**
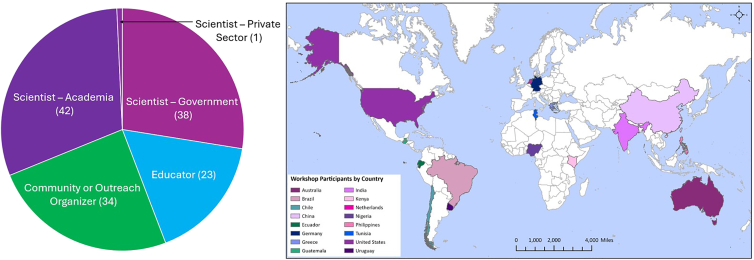
Workshop participants by sector (left) and country (right) (Left) Number of workshop participants split by the sector they represent. Note that the “educator” category refers to pre-college education only, and “community or outreach organizer” includes individuals working at nonprofit organizations, community or educational outreach facilitators, and GLOBE specialists. Due to the interdisciplinary nature of many attendees’ work, many are listed in more than one category. (Right) World map with countries where GLOBE workshop participants reside.

The workshop was attended by over 100 virtual participants from 16 countries and included 18 presentations and six discussion sections. These sessions brought scientists, educators, and community members together into a community of practice to discuss best practices for PM_2.5_ sensor quality assurance and quality control (QA/QC), metadata, satellite intercomparison, and community and educational use. The percentage of attendees from each of these sectors and the geographic locations where their projects are based are shown in Figure 1; note that many of the attendees conduct interdisciplinary work and thus were counted in more than one category. While most scientists who participated in the workshop were US based, many educators and GLOBE program coordinators conduct LCS work internationally, expanding the perspectives represented here. The full meeting agenda can be found in the appendix. No financial compensation was offered to workshop participants or presenters.

**Figure 2:**
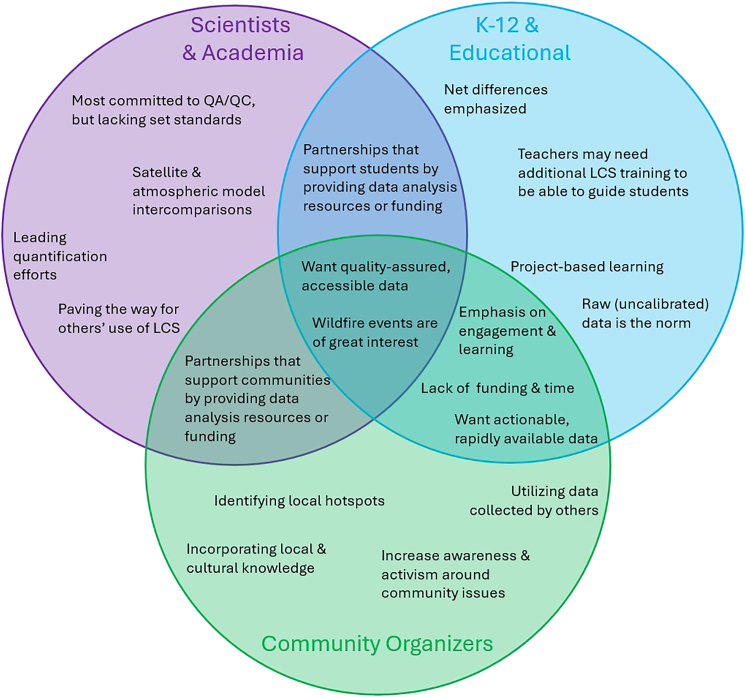
Venn diagram of stakeholder groups’ characteristics and needs

Consensus was reached on the need for data standardization, with barriers to access including end users’ ability to locate and interpret data. However, the group agreed that best practices vary by use case. Here, we aim to summarize these presentations (labeled as “overview” sections) and subsequent discussions to maximize scientific benefit from the immense network of global PM_2.5_ sensors, which are currently underutilized despite their global presence. Scientists, community organizers, and educators alike can consult this work for best practices. The status quo of each of these groups is shown in Figure 2; each of the subsequent sections will explore these characteristics in more detail.

## The current landscape of low-cost PM_2.5_ sensors

### Overview

Presenters laid the foundation of current efforts and trends in the PM_2.5_ sensor space, spanning long-established ground monitoring networks and recent calibration and harmonization efforts. The meeting began with a presentation on the recent World Meteorological Organization report, “Integrating Low-cost Sensor Systems and Networks to Enhance Air Quality Applications,” which set the stage for the current state of the art, challenges, and achievements in the LCS space.^10^ The report serves as a guide for both new sensor users looking to learn best practices and seasoned practitioners exploring new application areas.

Speakers also covered the derivation and application of generalized calibration tools, namely the US Environmental Protection Agency (EPA) PurpleAir PM_2.5_ US-Wide Standard Correction,^11^ and Wildfire Smoke Correction,^12^ which have been widely adopted for correcting raw PurpleAir measurements. A related study evaluated degradation of the PurpleAir sensors, finding that the average bias of measurements increased dramatically after 3.5 years of operation. Sensors also fared worse over time in hot and humid regions.^13^

QA/QC is another pressing topic^14^ to ensure that LCS data are both accessible and reliable. A recent review of LCS unification efforts^15^ led to the creation of NASA’s LCS Harmonization Database, an online archive that provides LCS data in a standardized data format with QA labels,^16^ which aims to ensure that scientific end users can find data of sufficient quality for their desired applications.

### Barriers to utilizing LCS and their data

Workshop speaker Ron Cohen noted that LCSs are “low capital cost, but measurements using LCSs are only low cost if labor costs are driven lower as well,” and these sentiments were echoed in the discussion session. While upfront hardware costs may be minimal, the labor needed to calibrate and upkeep an LCS network can be significant. Particularly for educational and community projects, the lack of ongoing funding for labor is a large barrier to maintaining a well-calibrated network.

Workshop participants cited the largest barriers to utilizing pre-existing LCS data generally as a lack of standard data and metadata formats, data accessibility issues including proprietary data and paywalls, and unclear or non-standard QA/QC procedures. Participants also noted a disconnect between scientific and community or educational projects. Data collected by communities are not typically regarded as being of scientific quality due to these QA/QC issues, while conversely, more involved analyses can be inaccessible to community members.

To remove these barriers, the group called for funded efforts to standardize LCS data and metadata, with efforts at the government or international level bearing the most legitimacy and potential for widespread adoption, with programs like AQ-SPEC^17^ in the US and MCERTS^18^ in the UK paving the way for LCS standards. Additionally, greater transparency in calibration and QA/QC procedures from both research institutions and companies would allow for broader usage, since end users cited deciphering disparate data formats as one of the main barriers to entry at present. Improved harmonization among disparate sensor networks would not only increase data accessibility but also awareness of many pre-existing networks, as end users cited not knowing where to find relevant data as another major issue.

### Calibration and QA/QC procedures

Protocol development is crucial for reproducibility and for matching end users with appropriate datasets. LCS data used for scientific purposes require standardized QA/QC procedures, protocols for determining uncertainty, best practices for consistent sensor siting, and a means of accounting for sensor drift, issues, and outliers. Standardized practices should also recognize that calibration needs will be different during large emission events, such as the EPA’s “extreme” wildfire smoke correction.^12^ Practitioners in the scientific community look to governmental bodies such as the US EPA for guidance and note that tools such as the AirSensor R package could be useful for standardizing data for scientific purposes.^19^

In educational contexts, calibration requirements are generally lax. Since net differences rather than quantification are often stressed, data producers should provide disclaimers on what the data can or cannot represent. Having students explore how to make their data reproducible on a larger scale could be a beneficial learning exercise but is generally not required for their intended use case.

Similarly, for community sensor usage, QA/QC requirements may be relaxed in comparison to scientific applications. Qualitative comparisons can help identify local issues (e.g., community hotspots), but quantitative evidence is often needed to prompt legislative action or to determine health effects. However, community LCS studies can be an important first step in drawing attention to a local issue. Providing training and financial support would also be necessary for community projects to achieve higher data quality. At present, a scientist championing a community data project is the most likely pathway to higher quality data.

### Public perceptions of LCSs

Discussions on calibration procedures revealed that the public tends to have relatively high confidence in any sensor data, regardless of whether it was calibrated. Where regulatory monitoring is insufficient, people are more likely to trust sensor readings at face value. Air Quality Index (AQI) metrics, which are calculated differently by different entities, can also lead to inconsistencies and confusion among the public. Participants also noted that independent data usage (e.g., community members seeking out LCS data to inform their own behaviors) is typically highest during large emission events such as wildfires. Tension was noted in these sessions between scientists, who are the primary developers of calibrations, and community members and educators, who discern that data are more actionable when disseminated quickly rather than “waiting” for QA/QC to be applied.

In educational settings, raw sensor data are primarily used to demonstrate qualitative differences; calibration is often considered too confusing or time-consuming to engage younger students in. Among researchers, calibration literacy is the highest, as is mistrust of uncalibrated or improperly calibrated data.

## Educational and participatory projects

### Overview

Presenters in this session focused their outreach efforts on students of all ages to increase knowledge of local AQ issues and data literacy. Various education outreach efforts at Xavier University engage their community in PM_2.5_ measurements through student construction of their own sensor packages, LCS teacher trainings, and community workshops.^20^ The Riverside Air Monitoring Project equipped high school students with PurpleAir and QuantAQ sensors, in addition to learning resources, to complete self-defined research projects. Learning outcomes were strongest for students who had full access to educational resources,^21^ and teacher focus groups were beneficial in identifying program strengths and weaknesses. Outside of the traditional classroom setting, a 2-week summer program for middle schoolers guided participants to visualize PurpleAir and Atmo Tube data to improve their data literacy. Likert-style questions revealed learning outcomes from the program,^22^ and the open access learning materials were released to enable greater reach. Another strong community-university partnership sought to examine PM_2.5_ exposure at schools in Utah, from which results were analyzed in relation to student demographics.^23^

### Improving relationships between scientists and communities

Community participation with LCS can vary, from individuals accessing data collected near them to actively participating in the deployment and analysis processes. When scientists and communities actively partner, sustained communication is crucial to ensuring community buy-in and data utility. Best practices might include ensuring the community has ownership of both the sensors and their data, providing data and analysis tools (see LCS data access and tools for recommended resources), listening sessions to incorporate local and cultural knowledge, and opportunities for the community to engage with the broader scientific community and share their findings.

### Data aggregation in community projects

Within community or advocacy groups, bridging the gap between disparate sensor networks is critical as many groups may not be aware of each other’s related efforts. Data aggregation platforms could broker these budding relationships between sensor networks with similar goals or in neighboring locations. Open access sensor data with consistent metadata would ensure ease of cross-community interactions.

## Fire monitoring techniques

### Overview

LCS networks can be impactful for plume tracking and modeling in urban and rural environments alike. The Berkeley Environmental Air quality & CO_2_ Network (BEACO_2_N) sensors, which have approximately 2 km coverage across parts of the San Francisco Bay Area, were used to track plume dilution and estimate emissions from a small urban fire.^24^ In Utah, another sensor network has been deployed to supplement large regulatory monitoring gaps across the state. The LCSs were utilized in a Gaussian model to provide highly resolved PM_2.5_ maps, revealing spatial differences in PM_2.5_ concentrations that were not apparent previously.^25^ At the intersection of community and scientific applications, PurpleAir sensors were placed near prescribed burning sites in Kansas, USA, with the help of citizen scientists. The LCSs were used to identify smoke-impacted days in the region, which were then investigated further using satellite-derived products,^26^ while community outcomes included different priorities between urban and rural survey responders.^27^

Recognizing the need for community visualization tools during wildfire events, the EPA and other federal agencies launched the AirNow Fire and Smoke Map,^28^ which includes data from nearly 17,000 non-regulatory PM_2.5_ sensors. The EPA “extreme” smoke correction described in barriers to utilizing LCS and their data is applied to the data, which the public can use to make informed decisions about their health during smoke events.^29^ The greater spatial coverage achieved by including LCSs on the map has been a boon, with the site receiving millions of page views during wildfire events.

### PM_2.5_ sensor limitations during wildfire events

During large wildfire events, larger particles (>10 μm) and ash are typically emitted but can be difficult to measure with LCSs, many of which target smaller particles (2.5 μm). Certain sensors, such as the Alphasense optical particle counters (OPCs), may perform better for larger particles. Participants also noted that most PM_2.5_ sensors exhibit a bias post-wildfire, potentially from clogging.

### Use cases of LCSs during wildfire events

Beyond the use cases described by the presenters, participants noted that LCSs are often utilized for personal exposure assessment during fires, with resources like the EPA’s Fire and Smoke Map receiving the most views during wildfire events. Comparisons between indoor and outdoor sensors in an area can inform end users on how much smoke is infiltrating indoors.^30^^,^^31^ LCSs have also been used to correlate smoke exposure to emergency room visits.^32^^,^^33^ Specific health effects have also been studied,^34^^,^^35^ although participants note that the lag between an event occurring and the availability of scientific literature often renders these findings non-actionable.

## LCSs as a complement to remote sensing

### Overview—Translating satellite AOD to surface PM_2.5_

Estimating surface PM_2.5_ concentrations from satellite-derived aerosol optical depth (AOD) measurements has traditionally relied on chemical transport models,^36^ but the rise of LCSs has afforded increased opportunities for ground-based validation. During a wildfire event in California, corrected PurpleAir PM_2.5_ measurements were found to correlate well with satellite AOD estimates.^37^ Another research group used a geographically weighted linear regression model to relate ground-based PM_2.5_ measurements to satellite-derived AOD estimates.^38^

Using LCSs specifically, including PurpleAir data alongside regulatory surface measurements, satellite AOD, and meteorological model data, improved a model’s effectiveness.^39^ In another approach, LCS PM_2.5_ and satellite AOD were utilized in a regression model alongside chemical model data to calculate PM_2.5_ in a multi-step process.^40^ Additionally, the International Data Fusion System was developed to estimate daily surface PM_2.5_ measurements at 1 km spatial resolution in Africa.^41^ Continued work with this method has yielded a prototype sub-city scale forecasting system.^42^

Ground-based remote sensing, including NASA’s Aerosol Robotic Network (Aeronet), has also been used to validate satellite AOD,^43^^,^^44^ and co-located LCSs have been impactful in translating AOD measurements to surface PM_2.5_. An ongoing pilot study of co-located LCSs and Aeronet aims to better characterize the spatiotemporal variability between surface PM_2.5_ and AOD.^45^ A neural network model has also been utilized to fuse Aeronet-constrained satellite AOD estimates with additional model- and ground-based data to generate PM_2.5_ surface estimates, which appear highly consistent with reference measurements, even under extreme conditions.^46^

Two presentations also covered validation efforts involving LCS for recently launched and upcoming AQ satellite missions. Information on the NASA Tropospheric Emissions: Monitoring of Pollution (TEMPO) satellite, which launched in 2023 and provides hourly observations of the continental United States,^47^ included preliminary particulate matter (PM) use cases during wildfires and dust storms. In preparation for the launch of the Multi-Angle Imager for Aerosols (MAIA) satellite in 2026, a ground-based PM_2.5_ network consisting of low- and middle-cost AQ sensors were deployed across Ethiopia to establish baseline concentrations in the region. These measurements will be used to validate and model daily PM_2.5_ readings from MAIA and, in the meantime, have been used to validate model estimates.^48^

### Considerations for using LCSs alongside satellite data

In addition to the modeling and forecasting applications presented in this session, LCSs can be used to validate ground-based estimates derived from satellites.^49^ Conversely, satellite data can also be used to validate sensor readings, especially in areas with sparse regulatory monitoring, although less common at present.

The complementary strengths and weaknesses of LCSs and satellite data make them great companion tools. While sensors have a higher temporal resolution than satellites, their spatial resolution varies. Satellites have high spatial coverage, but latency is often an issue. Together, satellites can fill gaps in sparse ground-based networks, and sensors can inform on sub-pixel trends. However, the data quality and access issues plaguing LCSs as described in barriers to utilizing LCS and their data remain a detriment, despite the sensors being more accessible than satellite data. Practitioners recommend ensuring proper QA/QC procedures for LCSs before considering them in any satellite work.

Other considerations include how fine a mesh of LCSs is needed for meaningful intercomparison with satellites, which varies by pollutant and application. Further, multiple LCSs placed within a single satellite pixel (typically 3.5 km or greater) have utility in sub-pixel and pixel boundary validation. Practitioners also noted that global models are difficult to achieve given large variations in PM_2.5_ and AOD, which also makes it difficult to scale smaller models to larger areas. To this end, existing atmospheric models can be employed to estimate the AOD-PM_2.5_ relationship in different regions, but model resolution and biases are still limiting factors.

### Barriers to using LCSs and satellite data jointly

Most participants cited a lack of technical knowledge or computational capacity as the main barrier to incorporating satellite data with their LCS data, which a 2024 assessment corroborates.^50^ Interpreting satellite data is beyond the scope of most educational and community projects. Further, at present, most satellite and LCS joint research is being conducted by scientists in wealthy countries, even if the research is based in low- and middle-income countries (LMICs).

## Recommendations for PM_2.5_ data collection and harmonization efforts

### Key takeaways for each stakeholder group

**Figure 3:**
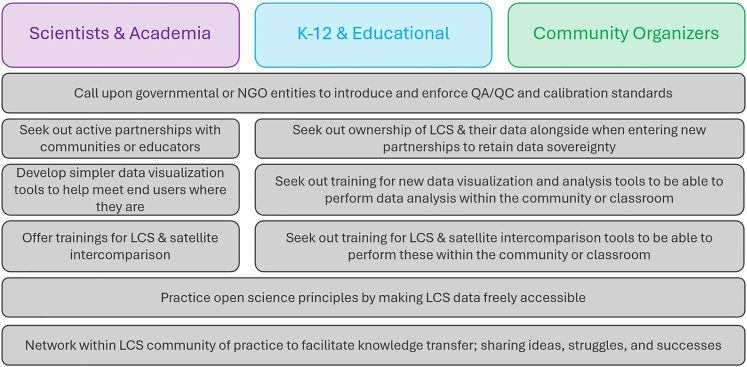
Key recommendations for each stakeholder group

The key recommendations based on the discussions held during this workshop can be found in Figure 3. No one entity is solely responsible for greater synthesis and cooperation between groups; the effort must be shared, with each group playing a distinct role. Scientists should expect to lead the charge in developing new data analysis and satellite intercomparison tools but may need to be willing to simplify them to encourage wider adoption. Educators and community organizers should seek out these trainings and be willing to challenge themselves, while being mindful of data sovereignty and growing their own capabilities. All three should be held responsible for making their data freely available, calling for and adhering to QA/QC standards, and communicating with the other groups. Each of these recommendations will require compromise; a balance must be struck between scientific rigor and accessibility as scientists pass analysis tools along to the other end-user groups. Likewise, developing frameworks where QA/QC and calibration can be applied rapidly could address scientists’ concerns while still honoring end users’ needs for real-time or near-real-time data during large emission events. By creating a united front, these groups have greater chances at success in meeting their shared goals.

### PM_2.5_ measurement techniques

**Figure 4:**
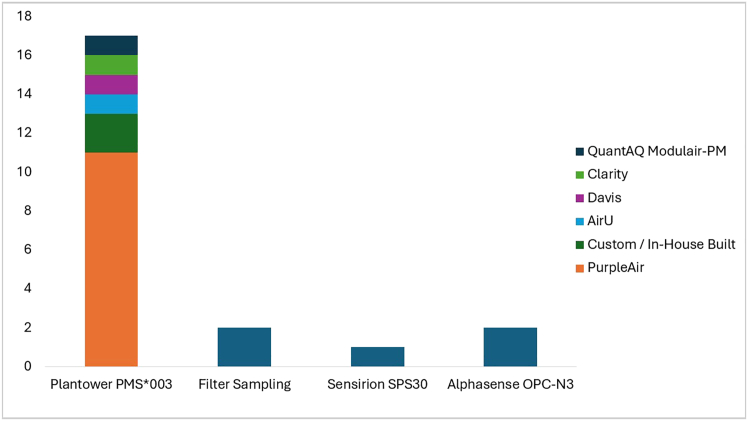
Histogram of low-cost PM_2.5_ sensors utilized by workshop speakers, with the most widely mentioned split into different sensor packages utilizing the sensor Sensor models in the Plantower PMS∗003 line include PMS5003, PMS6003, PMS1003, and PMSA003.

Although the speakers employed many different PM2.5 sensor packages, Figure 4 demonstrates that the vast majority utilize the same brand and type of sensor from the PMS∗003 line, which studies have shown perform similarly.^51^ This finding is relevant to the importance of standardizing data processing and archiving procedures; LCS data from the exact same or very similar sensors may be presented and processed in very different ways at present. Streamlined workflows, which treat all sensors the same, could make working with sensor data from different platforms much easier, given that they are functionally the same but currently treated differently.

Relative humidity is known to have a significant effect on the viability of most low-cost PM sensors,^52^ so a humidity correction should typically be applied. Sensor performance can also degrade over time, with most only exhibiting peak performance for 1–2 years,^53^ so frequent replacement of sensors should also be budgeted for. PM sensors can also become “clogged” with debris and insects, which participants recommended clearing with compressed air periodically.

### LCS data access and tools

Scientists requested a suite of GitHub tools and API access to streamline the process of downloading LCS data and noted that frameworks like the EPA’s Remote Sensing Information Gateway (RSIG) can make it easier to integrate satellite and LCS data and thus increase their usage of the dataset.^54^ Conversely, community members and educators noted that they would prefer simpler tools. Some found GLOBE’s pre-existing data visualization system to not be user-friendly enough or did not know where to find atmospheric data on the site. Community members requested simple tools for understanding local concentrations on their own, including auto-generated time-series plots of LCS data, and a means of comparing their LCS data to other local data, ranging from other LCSs to satellites. While many tools like this may already be freely available online, not all community members knew where to find them.

In terms of pre-existing data visualization and analysis tools, community members and educators tended toward online maps (AirNow and PurpleAir). Educators reported relying on tools such as the Common Online Data Analysis Platform (CODAP) and Jupyter notebooks, requesting “no experience necessary” platforms that would allow them to generate heatmaps and time-series plots with ease. Scientists’ preferred tools included ArcGIS online, NASA WorldView, and NASA’s STELLA data viewer, reinforcing that different audiences will require different tools.

### Encouraging satellite intercomparison with LCSs

To mitigate the barriers preventing most from incorporating satellite data into their LCS analyses, participants suggested training courses, especially for end users in LMICs, to help demystify satellite data. Pre-existing resources like NASA’s Applied Remote Sensing Training Program (ARSET) and GLOBE Watercooler discussions could be utilized with new content specifically targeting the integration of satellites with LCSs. Bespoke web tools to make intercomparison more user-friendly were also requested. Participants noted these may be especially valuable in LMICs, especially if local support was included in the form of both language translation and funding.

In educational settings, project-based learning approaches could make utilizing satellite data more accessible to students and early-career learners by providing hands-on training and emphasizing real-world applicability and skills. Participants noted that literacy in this area is low even among teachers, so teacher-training programs could be an impactful first step. Opportunities to collaborate more closely with scientists could also help foster engagement. Increased integration between these low- and high-cost tools also has the potential to bring more perceived legitimacy and awareness to AQ issues at the local level.

### Engagement and community building

Working with communities to ensure that sensor deployments are optimal for uncovering key pollution sources could help produce the highest possible mutual benefit from the data. Ensuring that communities can see the value add of their data in both the scientific community and their own lives is key for engagement.

To keep students engaged, project-based learning was emphasized for its real-world applicability.^55^^,^^56^ Ensuring that calibration requirements are simple enough for students to understand could help retain students’ interest. Student engagement and confidence are typically stronger in in-person activities as compared to virtual learning,^57^ further emphasizing the role of teachers and the classroom environment. Networking within the LCS community of practice can enable collaborations on project design, data sharing, and analysis. For instance, the GLOBE feature, which allows intercomparison among schools, is popular with students, and friendly competition among which schools produce the most data has incentivized students to remain active in the program.

### Broader implications

As discussed in public perceptions of LCS and use cases of LCS during wildfire events, the public may implicitly trust LCS data enough to make personal decisions based on it during wildfire or other extreme AQ events, but higher quality data and more complex analyses are still largely dependent on scientists. Scientists in the LCS space cannot each manage thousands of sensors, and end users with finite resources and technical knowledge cannot be expected to maintain their sensors to the same extent as paid professionals. Beyond the recommendations listed here, which mainly pertain to individual community and educational projects interfacing with scientists, standards and protocols for sensor calibration and quality would likely need to be adopted by governmental or international institutions to foster compliance within the broader LCS community. Scientists should aim to develop procedures and tools with the feedback of end users to ensure usability but rely on larger entities to encourage or enforce broader participation.

The need for LCS data to be of the highest quality possible and available for free to the public is not only a pain point for each of the three sectors presented here but is also essential to the continued growth and use of LCSs as a whole, due to their function as a democratic tool for bringing AQ information to areas that lack sufficient regulatory monitoring. Thus, it will not only take cooperation among these stakeholders but also likely intervention from larger, all-encompassing government or intergovernmental entities to implement these methods that apply to all three. Increased cooperation between these groups should lead the way for the specific types of regulations and standards needed. If governments cannot provide sufficient regulatory-grade monitoring, helping lead the way for these tools that help fill gaps in their own networks should be expected.

### Conclusions

The NASA GLOBE PM_2.5_ Low-Cost Sensor Workshop presented a unique opportunity for community leaders and educators to voice their reflections on presentations from the scientific community covering hot-button topics in PM_2.5_ sensing, offering insight on if and how the state of the art is being received and implemented by non-scientific audiences. Likewise, several presentations from educators on best practices for student learning and retention also allowed scientists to chime in with suggestions on producing higher quality data. In general, scientists need to meet LCS end users where they are and address the underlying reasons for end users not adopting more rigorous scientific practices to progress forward together. In return, educators and community members will need to challenge themselves with these new trainings and tools to eventually grow more capability and independence. These burdens could be lessened with stronger guidelines and tools from governmental organizations such as the NASA GLOBE program, providing more support for citizen scientists to collect better LCS data.

Recommendations for calibration include striking a balance between simplicity and ensuring data quality appropriate for expected applications. Data hosting and visualization tools should be created with non-scientific end users in mind, ensuring user-friendliness and including gateways to satellite data to encourage more users to consider their measurements in the larger AQ context. Overall, shaping LCS deployments and tools in line with community feedback can help bridge the gap between scientists and the broader community of practice, in turn maximizing use and efficacy of the thousands of pre-existing sensors.

### Materials availability

Workshop presentations and full notes from the discussion sections are available upon request; please contact the corresponding author.

## Acknowledgments

This work was funded by NASA’s GLOBE program, Mission Earth: Fusing GLOBE with NASA Assets to Build Systemic Innovation in STEM Education (award no. NNX16AC54A). Many thanks to our workshop presenters and participants.

## Author contributions

Conceptualization, M.P.; investigation, K.O., J.M., M.P., K.C., F.M., and S.M.; project administration, M.P., K.C., F.M., and S.M.; formal analysis, K.O.; writing – original draft, K.O. and J.M.; writing – review and editing, K.C. and F.M.

## Declaration of interests

The authors declare no competing interests.
